# Racial and socioeconomic disparities in long term survival after surgery and radiation for spinal cord hemangioblastoma

**DOI:** 10.1038/s41598-025-13330-7

**Published:** 2025-08-21

**Authors:** Abdul Karim Ghaith, Xinlan Yang, Joshua Weinberg, Shaan Bhandarkar, Taha Khalilullah, Abdel-Hameed Al-Mistarehi, Khaled Zaitoun, Meghana Bhimreddy, Arjun K. Menta, Antony Fuleihan, Kamel Chebaro, Jawad Khalifeh, Andrew Grossbach, Nicholas Theodore, Daniel Lubelski

**Affiliations:** 1 School of Medicine, Department of Neurosurgery, Johns Hopkins University, 600 N. Wolfe Street / Meyer 5-181, Baltimore, MD 21287 USA; 2 Department of Neurosurgery, School of Medicine, Ohio State University, Columbus, OH USA; 3 Sidney Kimmel Medical College, Thomas Jefferson University, Philadelphia, PA USA; 4 Faculty of Medicine, Jordan University of Science and Technology, Irbid, Jordan; 5 School of Medicine, Humanitas University, Pieve Emanuele, Milan, Italy

**Keywords:** Spinal cord hemangioblastomas, Socioeconomics, Racial disparities, Survival machine learning models, Cancer, Neurology, Oncology

## Abstract

Spinal cord hemangioblastomas are rare, benign, intradural tumors that, despite their nonmalignant histopathology, can lead to substantial neurological morbidity. While disparities in outcomes based on race and socioeconomic status have been well-documented in other spinal tumor populations, their role in spinal cord hemangioblastoma remains poorly understood. In this study, we utilize the National Cancer Database (NCDB) to evaluate the influence of race, socioeconomic factors, and healthcare access on survival outcomes in patients with spinal cord hemangioblastoma. Additionally, we explore the utility of machine learning–based survival models to improve individualized risk prediction and to identify key clinical and sociodemographic determinants of long-term survival. Patients diagnosed with spinal cord hemangioblastoma were identified from the National Cancer Database (NCDB) using ICD-O-3 histology and topography codes. Demographic, socioeconomic, and clinical variables were compared across racial groups (White, Black and Asian). Long-term overall survival (OS) was defined as survival beyond 10 years. Kaplan–Meier and multivariable Cox regression analyses were used to evaluate survival outcomes and identify independent predictors of mortality. Tumor size was stratified using the cohort-wide mean (62.2 mm) for interpretability. Temporal trends in racial distribution and surgical technique (open vs. MIS) were assessed using Mann–Kendall trend testing. Gradient Boosting Survival, Cox proportional hazards, and Random Survival Forest models were developed and validated for mortality prediction. The best-performing model was interpreted using SHAP analysis. A total of 716 adult patients with spinal cord hemangioblastoma were analyzed, with the majority being White (83.7%), followed by Black (12.3%) and Asian (4%). Significant differences were observed across racial groups in age, insurance status, income quartiles, and comorbidity scores, though sex distribution and facility type utilization were comparable. Most patients were treated at academic centers, and surgery alone was the predominant treatment modality, with no racial disparities in extent of resection or use of radiation. Kaplan–Meier analysis showed significantly higher 10-year and long-term mortality in White patients; however, race was not an independent predictor in multivariable Cox regression, where increased age, higher CDCC scores, urban residence, and treatment at comprehensive community cancer centers were associated with worse survival. Surgery, with or without radiation, was protective compared to radiation alone. Temporal analysis showed stable racial distribution and minimal uptake of minimally invasive surgery from 2010 to 2017. The Gradient Boosting Survival model achieved the highest predictive performance (AUC = 0.8214; C-index = 0.7817), with age, facility type, and comorbidity burden identified as the strongest predictors of mortality in SHAP analysis. A publicly available web-based calculator was developed based on this model to provide individualized survival estimates. Racial and socioeconomic disparities were associated with differences in clinical outcomes on univariate analysis. However, race and insurance status were not independent predictors of mortality in multivariable-adjusted models. This suggests that the observed survival differences may be explained by confounding factors, such as comorbidity burden, treatment modality, or access to specialized care. Notably, poorer survival was independently associated with treatment at Comprehensive Community Cancer Programs and with higher comorbidity scores, underscoring the importance of ensuring equitable access to high-volume, specialized centers. Lastly, the Gradient Boosting Survival model enhanced mortality risk prediction by incorporating both clinical and socioeconomic variables, supporting its potential utility in guiding targeted interventions to improve long-term outcomes.

**Supplementary Information:**

The online version contains supplementary material available at 10.1038/s41598-025-13330-7.

## Introduction

Spinal cord hemangioblastomas are rare, benign vascular tumors of the central nervous system (CNS), accounting for approximately 2–10% of all spinal cord neoplasms. According to data from the Surveillance, Epidemiology, and End Results (SEER) database, the age-adjusted incidence of spinal cord hemangioblastomas is estimated at 0.014 per 100,000 individuals, making them the tenth most common intradural spinal tumor type.^1–4^ Despite their benign histology, these tumors can lead to significant morbidity due to their location within the spinal cord. A study utilizing SEER data reported a median survival of 27.5 months, with a 9% mortality rate over a 10-year period.^5^

While the majority of spinal cord hemangioblastomas are sporadic, approximately 20–30% are associated with von Hippel-Lindau (VHL) disease, an autosomal dominant hereditary syndrome caused by mutations in the VHL tumor suppressor gene. Patients with VHL disease often present with multiple CNS hemangioblastomas and other neoplasms, necessitating careful surveillance and management.^2,4^

Surgical resection remains the primary treatment modality for spinal cord hemangioblastomas, offering the potential for cure. However, due to the tumor’s proximity to critical spinal cord structures, surgery carries a significant risk of postoperative neurological deficits. Although advancements have been made in radiation therapy, its efficacy in treating spinal cord hemangioblastomas remains a subject of debate.^1,6^

Beyond clinical considerations, real-world outcomes are significantly influenced by socioeconomic factors. Previous studies have demonstrated that patients from lower socioeconomic backgrounds often face disparities in treatment access and outcomes for spinal tumors.^7,8^ For instance, research indicates that Black and Hispanic patients are less likely to undergo surgical intervention, experience higher postoperative complication rates, and endure longer, more costly hospital stays. Additional barriers, such as financial hardship, limited education, and low health literacy, may further exacerbate these disparities. Despite these findings, the specific impact of race and socioeconomic status on outcomes in spinal cord hemangioblastoma remains poorly understood.^9^

Recent advancements in machine learning offer the potential to model these disparities more precisely. By integrating both clinical and socioeconomic variables, machine learning-based survival models can provide nuanced, individualized predictions of mortality risk, thereby enhancing our understanding of outcome determinants.^10,11^.

This study aims to investigate racial and socioeconomic disparities among patients with spinal cord hemangioblastoma using data from the National Cancer Database (NCDB). We compared socioeconomic variables among three racial groups (White, Black, and Asian) and assessed the primary outcome of long-term overall survival (OS), along with other clinical outcomes. Additionally, we developed a publicly accessible Gradient Boosting Survival model-based calculator to predict personalized mortality risk by incorporating socioeconomic factors.

## Methods

### Cohort selection

The NCDB, one of the largest U.S. cancer registries, includes nearly 34 million cases from over 1,500 hospitals accredited by the American College of Surgeons’ Commission on Cancer (CoC). Sponsored by the ACS and the American Cancer Society, the NCDB enables multi-institutional retrospective research.

We queried the NCDB for patients diagnosed and treated for spinal cord hemangioblastoma (ICD-O-3 histology code 9161) and filtered cases using the ICD-O-3 topographical codes for tumors located in the spinal cord (C72.0) and cauda equina (C72.1). Pediatric patients aged 20 or below were excluded. The study complies with the Health Insurance Portability and Accountability Act (HIPAA) and follows the Strengthening the Reporting of Observational Studies in Epidemiology (STROBE) reporting guideline. Our institutional review board (IRB) determined it to be exempt. Our IRB deemed it exempt, and informed consent was not required for de-identified data use.

This study was approved by Johns Hopkins University Institutional Review Board under a general study protocol (IRB#: CIR00110888) for analyses using NCDB data and was performed in accordance with the Declaration of Helsinki. Informed consent was waived by Johns Hopkins University Institutional Review Board.

### Patient demographics and disease characteristics

The cohort identified from NCDB was categorized based on race into White, Black, and Asian, and the demographic, socioeconomic, geographic, treatment, and clinical outcomes data were compared across racial groups. Variables analyzed included age at diagnosis, sex, facility type, insurance status, median income quartile, type of facility (defined by the CoC-accredited cancer programs), distance from home to facility in miles, Charlson-Deyo Comorbidity Classification (CDCC) scores, residential location (urban, metro, rural) and tumor size measured in the largest diameters in millimeters (mm). Facility type was categorized into Academic/Research Program, Community Cancer Program, Comprehensive Community Cancer Program, and Integrated Network Cancer Program based on the definitions defined in the NCDB database dictionary. To note, tumor size was initially treated as a continuous variable in millimeters. For improved interpretability, we also created a binary variable using the cohort’s mean tumor size (62.2 mm) as a cutoff. Patients were categorized into those with tumors ≤ 62.2 mm and > 62.2 mm, and this binary variable was used in multivariable Cox regression analysis.

Categorical variables were presented in frequency and percentage, and continuous variables were presented in mean and standard deviation.^12^ It is important to note that median income by ZIP code and residential location (urban, metro, rural) are area-level measures and may not accurately reflect an individual patient’s socioeconomic status. These variables are commonly used in large datasets such as the NCDB, but they are subject to ecological fallacy and should be interpreted with caution.

### Treatment characteristics

This study included adult patients (> 20 years) with spinal cord hemangioblastoma. Treatment modalities were summarized into surgery alone, radiation alone, or combined surgery and radiation. Extent of resection was categorized based on the NCDB variable RX_SUMM_SURGICAL_MARGINS. Gross Total Resection (GTR) was defined as code 0 (no residual tumor, i.e., all margins grossly and microscopically negative), while Subtotal Resection (STR) included codes 1, 2, and 3 (residual tumor present, either macroscopic or microscopic). These data are abstracted from operative and pathology reports; however, whether margin status was determined by surgical impression or imaging is not specified. Radiation modality and total dose were recorded. Neurological status was not available in the NCDB dataset. The database does not include detailed patient-level neurological assessments or standardized functional outcome scores such as motor/sensory deficits, bowel/bladder status, or scales like McCormick or Nurick grades.

### Primary and secondary outcome variables

The primary outcome for Kaplan–Meier survival analysis was long-term overall survival (OS), defined as survival from diagnosis to last follow-up, with long-term survival specifically referring to survival beyond 10 years. Secondary outcomes included 30-day readmission rates, short-term mortality at 30 days, 90 days, and 1-year, long-term mortality at 5 years.

### Statistical analysis

We performed univariate comparisons between the racial groups White, Black, and Asian. Categorical variables were analyzed using Chi-square tests or Fisher’s exact tests. In contrast, continuous variables were analyzed using independent samples t-tests for parametric data or Mann–Whitney U tests for nonparametric data. To approximate anatomical tumor location, we categorized tumors based on ICD-O-3 topography codes: C72.0 (spinal cord) and C72.1 (cauda equina). This binary classification was used to stratify patients into spinal cord and cauda equina groups, serving as a surrogate for spinal level involvement. We then performed a subgroup analysis comparing demographics, treatment characteristics, and overall survival between these two groups.

The primary outcome, long-term OS, was compared between groups using Kaplan–Meier survival curves and the Log-rank test, with statistical significance defined as *p* < *0.05*. The KM curves were limited to 120 months due to insufficient patients with survival data available beyond 10 years. A post hoc power analysis was conducted using the observed survival rates beyond 10 years between White and Black patients.

All statistical analyses were conducted using R Studio (*v4.4.2*) and Python (*v3.13.0*), adhering to STROBE reporting guidelines for observational studies. Risk factor analysis was performed using a multivariate Cox proportional hazards model by including age, sex, race, CDCC comorbidity scores, facility type, insurance status, home-to-facility distance, median income quartile, tumor size, primary tumor site (spinal cord vs cauda equina), intervention, and extent of resection as variables, with hazard ratios (HRs), 95% confidence intervals (CIs), and *p-values* reported for each. Tumor size was categorized dichotomously using the cohort-wide mean of 62.2 mm as the cutoff.

To assess temporal patterns in patient demographics and treatment strategies, we analyzed racial distribution and surgical technique trends over time. For racial composition, patients diagnosed between 2004 and 2017 were stratified by race (White, Black, Asian), and annual proportions were visualized using a stacked bar chart with the total number of cases per year overlaid. For surgical approach, cases diagnosed between 2010 and 2017 were categorized as having undergone open or minimally invasive surgery (MIS), and annual utilization was plotted separately. To test for monotonic trends in race distribution, patient volume, and surgical approach over time, we applied the Mann–Kendall trend test, a non-parametric method suitable for detecting directional trends in time-series data.

The dataset was randomly partitioned into a training set (80%) and a test set (20%) using stratified sampling to preserve the class distribution of the outcome variable (*Dead*). Within the training set, fivefold cross-validation was conducted to tune hyperparameters and assess internal model performance. To address class imbalance, the Synthetic Minority Oversampling Technique (SMOTE) was integrated within each cross-validation fold using pipeline-based implementation to avoid data leakage.

Three survival models were developed and evaluated: Cox Proportional Hazards (CoxPH) using the *lifelines* library, Random Survival Forest (RSF), and Gradient Boosting Survival (GBS) using *scikit-survival*. Hyperparameter optimization for GBS included tuning the number of estimators (*n_estimators*), learning rate, and tree depth (*max_depth*), while RSF tuning involved selecting the number of trees (*n_estimators*), minimum samples per split, and *max_features*. Grid search was used in conjunction with cross-validation to determine optimal model parameters.

After cross-validation, the best-performing model, Gradient Boosting Survival, was retrained on the entire training set using optimal hyperparameters and evaluated on the independent test set. Final model performance was assessed using Area Under the ROC Curve (AUC), Brier score, and Harrell’s Concordance Index (C-index).

To enhance model interpretability, SHapley Additive exPlanations (SHAP) analysis was applied to the test set predictions. This allowed for identification of the most influential predictors of long-term mortality (over 10 years) while ensuring interpretive validity by restricting the analysis to previously unseen data. Based on superior discrimination, calibration, and explainability, the GBS model was selected for deployment as the backbone of the web-based survival risk calculator. The calculator incorporates key clinical and socioeconomic input variables including age, sex, race, insurance status, facility type and location, CDCC score, treatment modality, tumor size, income quartile, and crowfly distance. It is intended for exploratory and research use to estimate individualized mortality risk.

## Results

### Patient demographics and socioeconomic characteristics

A total of 716 adult patients diagnosed and treated for hemangioblastoma were included in this study, with the majority being White (N = 599, 83.7%), followed by Black (N = 88, 12.3%) and Asian patients (N = 29, 4%) (Table 1). The mean age differed significantly across racial groups (*p* = *0.009*), with White patients being the oldest (50.2 ± 16 years), followed by Black (46.6 ± 14.8 years) and Asian (42.6 ± 14.1 years). Sex distribution was similar across groups (male: 52%, female: 48%; *p* = *0.184*). Facility utilization was comparable across races, with academic or research programs being the most frequently utilized (75.4%), followed by the Comprehensive Community Cancer Programs (14.5%) (*p* = *0.202*). Insurance status varied significantly (*p* = *0.008*): White patients were most prevalently covered by private insurance (65.1%) and Medicare (21.5%); while Medicaid was more common among Black (20.5%) and Asian patients (20.7%) compared to White patients (7.7%). Income quartile distributions also varied significantly (*p* < *0.001*): the highest-income quartile was most prevalent among White (46.1%) and Asian (51.7%) patients, whereas Black patients had the highest proportion in the lowest-income quartile (31.8%), exceeding two-fold higher than the White (10.0%) or Asian populations (17.2%).

**Table 1:** Demographics, Disease Characteristics and Clinical Outcomes of Patients with Spinal Cord Hemangioblastoma by Race.

	Total (N = 716) No. (%)	White (N = 599) No. (%)	Black (N = 88) No. (%)	Asian (N = 29) No. (%)	*p-value*
Age, mean (SD), years	49.4 (15.9)	50.2 (16.0)	46.6 (14.8)	42.6 (14.1)	**0.009**
Sex					0.184
Female	344 (48.0)	279 (46.6)	50 (56.8)	15 (51.7)	
Male	372 (52.0)	320 (53.4)	38 (43.2)	14 (48.3)	
Facility Type					0.202
Academic/Research Program	540 (75.4)	445 (74.3)	69 (78.4)	26 (89.7)	
Community Cancer Program	7 (1.0)	6 (1.0)	0 (0.0)	1 (3.4)	
Comprehensive Community Cancer Program	104 (14.5)	92 (15.4)	10 (11.4)	2 (6.9)	
Integrated Network Cancer Program	65 (9.1)	56 (9.3)	9 (10.2)	0 (0.0)	
Insurance Status					**0.008**
Private Insurance	457 (63.8)	390 (65.1)	51 (58.0)	16 (55.2)	
Medicare	149 (20.8)	129 (21.5)	15 (17.0)	5 (17.2)	
Medicaid	70 (9.8)	46 (7.7)	18 (20.5)	6 (20.7)	
Other Government	9 (1.3)	9 (1.5)	0 (0.0)	0 (0.0)	
Not Insured	31 (4.3)	25 (4.2)	4 (4.5)	2 (6.9)	
Median Income quartile					** < 0.001**
Highest	323 (45.1)	276 (46.1)	32 (36.4)	15 (51.7)	
High—Middle	167 (23.3)	147 (24.5)	12 (13.6)	8 (27.6)	
Middle—Low	133 (18.6)	116 (19.4)	16 (18.2)	1 (3.4)	
Lowest	93 (13.0)	60 (10.0)	28 (31.8)	5 (17.2)	
Residential Location					0.796
Metro	634 (88.5)	529 (88.3)	78 (88.6)	27 (93.1)	
Rural	6 (0.8)	6 (1.0)	0 (0.0)	0 (0.0)	
Urban	76 (10.6)	64 (10.7)	10 (11.4)	2 (6.9)	
Distance from home to facilities, Mean (SD), miles	53.2 ± 111.5	57.7 ± 119.9	28.8 ± 37.7	35.9 ± 59.1	0.053
Charlson-Deyo Comorbidity Classification (CDCC) scores					**0.043**
0	575 (80.3)	490 (81.8)	60 (68.2)	25 (86.2)	
1	90 (12.6)	69 (11.5)	18 (20.5)	3 (10.3)	
≥ 2	51 (7.1)	40 (6.7)	10 (11.4)	1 (3.4)	
Tumor Size, Mean (SD), mm	62.2 (72.5)	62.6 (69.7)	65.5 (96.2)	42.6 (33.5)	0.313
Treatment					0.432
Surgery alone	623 (87.0)	515 (86.0)	81 (92.0)	27 (93.1)	
Radiation alone	17 (2.4)	16 (2.7)	1 (1.1)	0 (0.0)	
Surgery with Radiation	76 (10.6)	68 (11.4)	6 (6.8)	2 (6.9)	
Surgical Techniques					0.551
Open	386 (95.5%)	311 (95.1%)	57 (98.3%)	18 (94.7%)	
MIS	18 (4.5%)	16 (4.9%)	1 (1.7%)	1 (5.3%)	
Na	312	272	30	10	
Extent of Resection					0.206
GTR	688 (98.4)	576 (98.8)	84 (96.6)	28 (96.6)	
STR	11 (1.6)	7 (1.2)	3 (3.4)	1 (3.4)	
Duration from diagnosis to surgery, Mean (SD), days	24.4 ± 55.0	23.8 ± 51.4	19.5 ± 40.1	52.0 ± 123.6	**0.018**
Primary Site					0.68
Spinal Cord	705 (98.5%)	590 (98.5%)	86 (97.7%)	29 (100%)	
Cauda Equina	11 (1.5%)	9 (1.5%)	2 (2.3%)	0 (0%)	
Radiation Therapy					0.172
Yes	93 (13.1)	84 (14.2)	7 (8.0)	2 (6.9)	
No	616 (86.9)	509 (85.8)	80 (92.0)	27 (93.1)	
Radiation Modality					0.382
Photon	60 (64.5)	54 (64.3)	4 (57.1)	2 (100.0)	
Brachytherapy	33 (35.5)	30 (35.7)	3 (42.9)	0 (0.0)	
Total Dose, Mean (SD), cGy	3968.0 ± 1740.3	3855.7 ± 1755.3	4890.0 ± 1571.4	4500.0 (NA)	0.374
*Clinical Outcomes*					
Follow-up Time, Mean (SD), months	69.0 (42.5)	68.7 (43.1)	72.7 (39.9)	64.5 (36.8)	0.598
Length of stay, Mean (SD), days	5.6 (6.5)	5.4 (6.3)	6.5 (8.1)	6.0 (4.3)	0.333
30-day Readmission rate	46 (6.4)	34 (5.7)	9 (10.2)	3 (10.3)	0.181
*Mortality rates*					
30-day	3 (0.5)	3 (0.6)	0 (0.0)	0 (0.0)	0.744
90-day	9 (1.4)	9 (1.7)	0 (0.0)	0 (0.0)	0.412
5-year	63 (8.8)	57 (9.5)	5 (5.7)	1 (3.4)	0.289
10-year	88 (12.3)	82 (13.7)	5 (5.7)	1 (3.4)	**0.034**
Long-term	94 (13.1)	88 (14.7)	5 (5.7)	1 (3.4)	**0.019**

GTR, gross total resection; STR, subtotal resection.

**Fig. 1:**
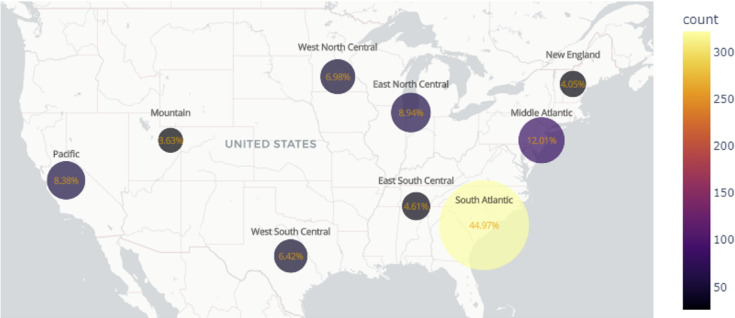
Geographic incidence of treated spinal cord hemangioblastoma cases across the United States between 2004 and 2017. The map was generated using Python (version 3.11.4) with the Plotly library (version 5.19.0) for interactive choropleth visualization. Regional percentages reflect the proportion of total national cases treated in each U.S. Census region. Software URL: https://plotly.com/python.

No significant differences were found in residential locations, as the majority of patients (88.5%) resided in metropolitan areas, followed by urban areas (10.6%) *(p* = *0.796*). White patients, on average, traveled the farthest to access facilities (57.7 ± 120 miles), followed by Asian (35.9 ± 59.1 miles) and Black patients (28.8 ± 37.7 miles), although statistical significance was not reached (*p* = *0.053*). In the analysis of geographic incidence across the United States, South Atlantic regions reported the largest number of cases (45%), followed by the Middle Atlantic (12%) and East North Central regions (9%) (Fig. 1).

### Clinical characteristics and treatment modalities

The CDCC scores differed significantly across racial groups (*p* = *0.043*). White (81.8%) and Asian (86.2%) patients had larger proportion of patients scoring 0 in CDCC compared to Black population (68.2%), whereas Black population had the highest prevalence of patients with higher CDCC score (≥ 2) (11.5%) over White (6.7%) or Asian (3.4%) populations. Tumor size did not differ significantly across groups *(p* = *0.313*), with a mean tumor size of 62.2 ± 72.5 mm in the study cohort (Table 1).

Surgery alone was the most used treatment across all racial groups. Radiation therapy, either alone or in combination with surgery, was less frequently employed, with no significant differences between groups. Among surgically treated patients, GTR was achieved in 98.4% of cases, with no racial disparities (White: 98.8%, Black: 96.6%, Asian: 96.6%; *p* = *0.206*). Radiation therapy was administered to 13.3% of the study population (*p* = *0.172*), with photon therapy being the most used modality across all groups (*p* = *0.382*). The total dose also did not differ significantly, with a cohort-wide average of 3968 ± 1740.3 cGy (*p* = *0.374*). Tumor location did not differ significantly across racial groups (*p* = *0.68*). The vast majority of tumors were located in the spinal cord across all races (White: 98.5%, Black: 97.7%, Asian: 100%), while cauda equina tumors were rare (White: 1.5%, Black: 1.7%, Asian: 0%) (Table 1).

### Clinical outcomes

The cohort was followed for approximately 69 months (Table 1). The mean length of stay (LOS) after surgery was 5.6 ± 6.5 days, with Black patients having a slightly longer LOS (6.5 ± 8.1 days) compared to others, though this difference was not statistically significant (*p* = *0.333*). Readmission within 30 days was uncommon across all groups, with White patients having the lowest rate (5.7%) compared to Black (10.2%) and Asian patients (10.3%) without reaching statistical significance (*p* = *0.181*). Short-term mortality was also rare, with 30-day and 90-day mortality occurring in 0.5% (*p* = *0.744*) and 1.4% (*p* = *0.412*) of patients, respectively. The 5-year mortality rate did not differ significantly across groups (*p* = *0.289*). However, 10-year and long-term (> 10 years) were significantly higher in White patients, nearly two-fold higher than the other two races at both time points (*p* = *0.034* and *p* = *0.019*, respectively).

**Fig. 2:**
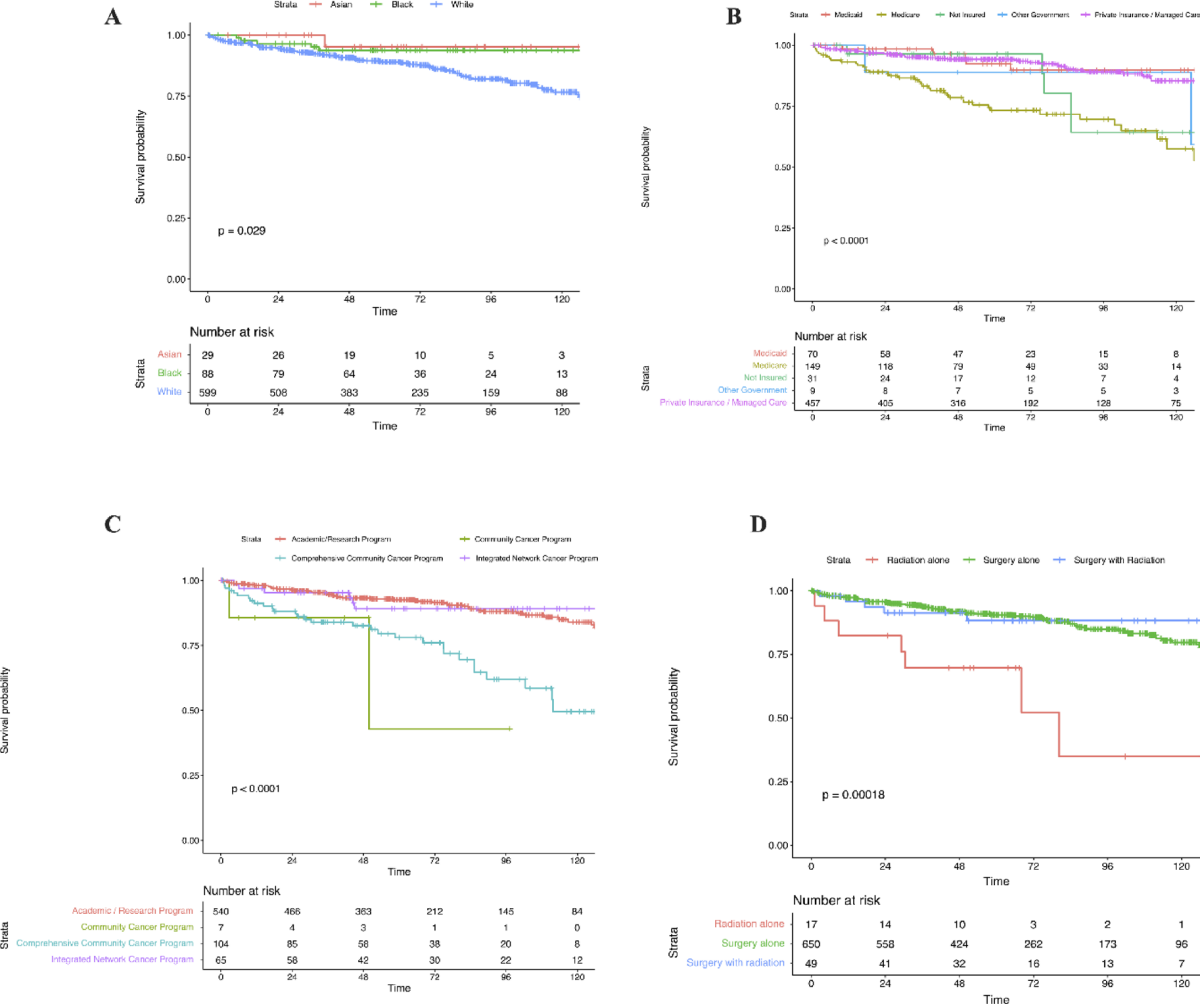
Kaplan–Meier Analysis of Overall Survival in Spinal Cord Hemangioblastoma Patients by (**A**) Race, (**B**) Insurance Status, (**C**) Facility Type, and (**D**) Treatment Type, with survival follow-up over 10 years.

KM survival analysis showed significant impact of patient characteristics on the long-term OS. While unadjusted analyses showed higher long-term mortality among White patients, this association did not persist in the multivariate Cox regression model (*p* = *0.029*). This underscores the importance of adjusting for clinical and socio-economic variables when evaluating demographic differences in survival outcomes. (Fig. 2A). Insurance status also influenced OS, with Medicaid, private insurance, and other government insurance associated with better survival compared to Medicare or being uninsured (*p* < *0.001*) (Fig. 2B). Patients treated at academic/research programs and integrated network cancer programs had the best OS, in contrast to patients treated in the Comprehensive Community Cancer Programs or the Community Cancer Programs (*p* < *0.001*) (Fig. 2C). For treatment type, patients treated with surgery alone or combined surgery and radiation had better survival compared to ones treated with radiation alone (*p* < *0.001*) (Fig. 2D).

Regarding our trend analysis, the racial distribution remained relatively stable across the study period, with White patients comprising the majority each year and no significant shifts observed in the proportional representation of Black or Asian patients (Mann–Kendall *p* = 0.21). Annual patient volume varied slightly but did not show a significant trend over time (*p* = 0.39). From 2010 to 2017, open surgery was consistently the predominant approach, while MIS was rarely utilized and did not show a statistically significant increase in use during the study period (Mann–Kendall *p* = 0.31). (Supplementary Fig. 1 A and B). A post hoc power analysis was conducted using the observed 10-year survival rates between White (14.7%) and Black (5.7%) patients. Based on a total sample size of 716 and an alpha level of 0.05, the estimated effect size (Cohen’s h = 0.305) yielded a power > 99.9%. This confirmed that the study was adequately powered to detect the observed survival difference between racial groups.

### Predictive modelling

**Fig. 3:**
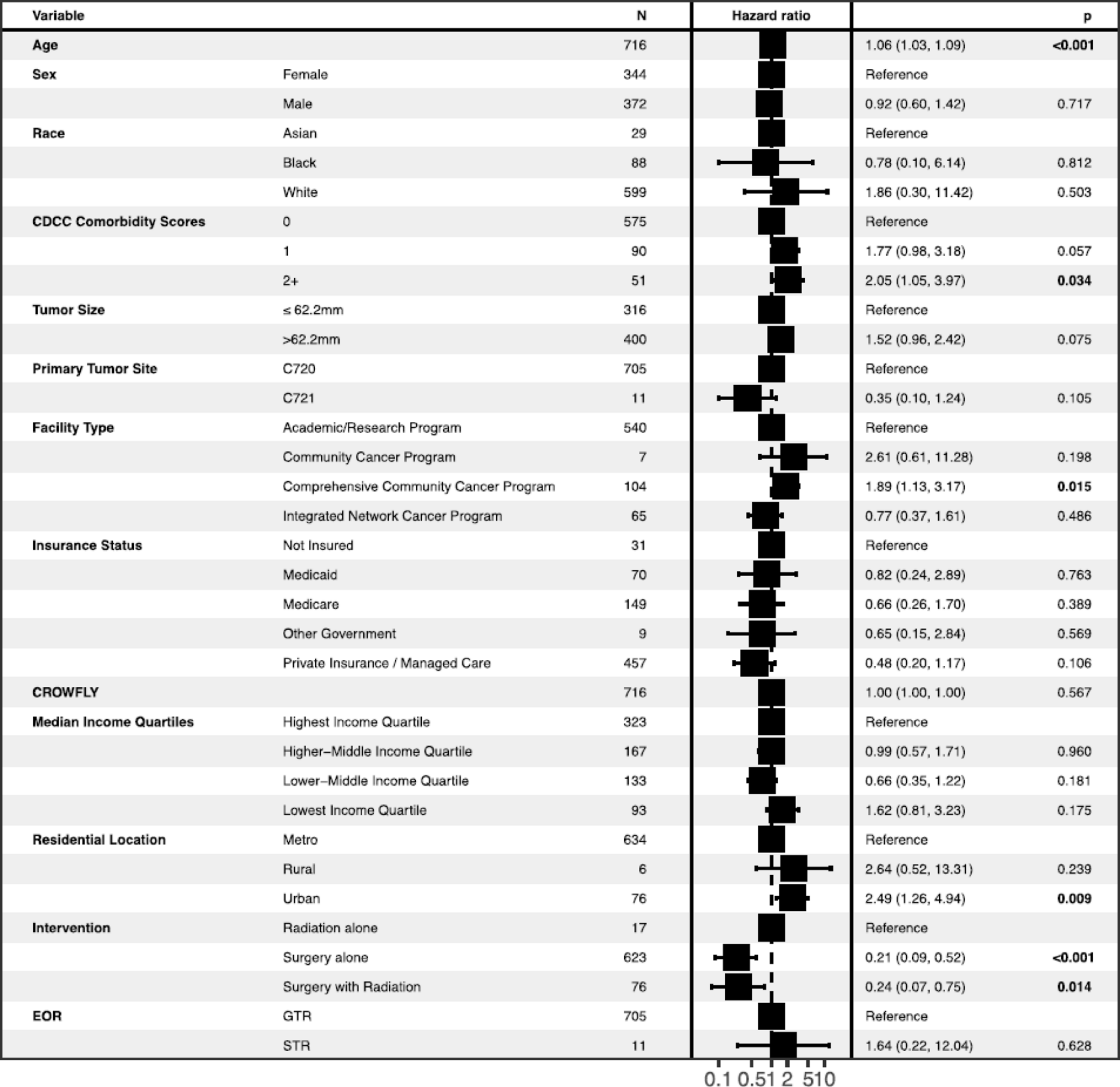
Multivariable Cox regression model showing adjusted hazard ratios (HRs) and 95% confidence intervals. These results reflect associations adjusted for all covariates included in the model. Statistically significant predictors (*p* < 0.05) are bolded for clarity. HRs represent multivariate-adjusted hazard risk estimates. Abbreviations: GTR, gross total resection; STR, subtotal resection.

The Cox proportional hazards model identified key risk factors for long-term mortality (Fig. 3). Higher age was linked to a significantly greater mortality risk (HR = 1.06, *p* < *0.001*). Increased CDCC scores were associated with higher mortality risk (scores 2 + : HR = 2.05, *p* = *0.034*). Tumor site was not significantly associated with mortality risk. Patients with cauda equina tumors demonstrated a trend toward lower mortality risk compared to those with spinal cord tumors, though this did not reach statistical significance (HR = 0.33, *p* = *0.073*). Larger tumor size (> 62.2 mm) was associated with a non-significant trend toward increased mortality compared to smaller tumors ≤ 62.2 mm (HR = 1.52, *p* = *0.075*). Patients treated at comprehensive community cancer programs (HR = 1.89, *p* = *0.015*) and those residing in urban areas (HR = 2.49, *p* = *0.009*) also had higher mortality risk. Compared to radiation alone, surgery alone (HR:0.21, *p* < *0.001*) and combined surgery with radiation (HR:0.24 *p* = *0.014*) were both associated with lower risk of mortality. No significant differences in mortality risk were observed across racial groups. Other factors, including sex, insurance status, home-to-facility distance, median income quartile, and extent of resection did not have a significant impact. The Multivariate Cox regression results for predictors of long-term OS for spinal cord hemangioblastoma patients are detailed in Supplementary Table 1, showing HRs, 95% CIs, and p-values for each covariate included in the model as represented in Fig. 3. While Fig. 2 presents unadjusted survival differences based on Kaplan–Meier curves and log-rank testing, Fig. 3 shows results from a multivariable Cox regression model, reporting adjusted hazard ratios accounting for all covariates in the model.

### Model performance and feature importance

**Fig. 4:**
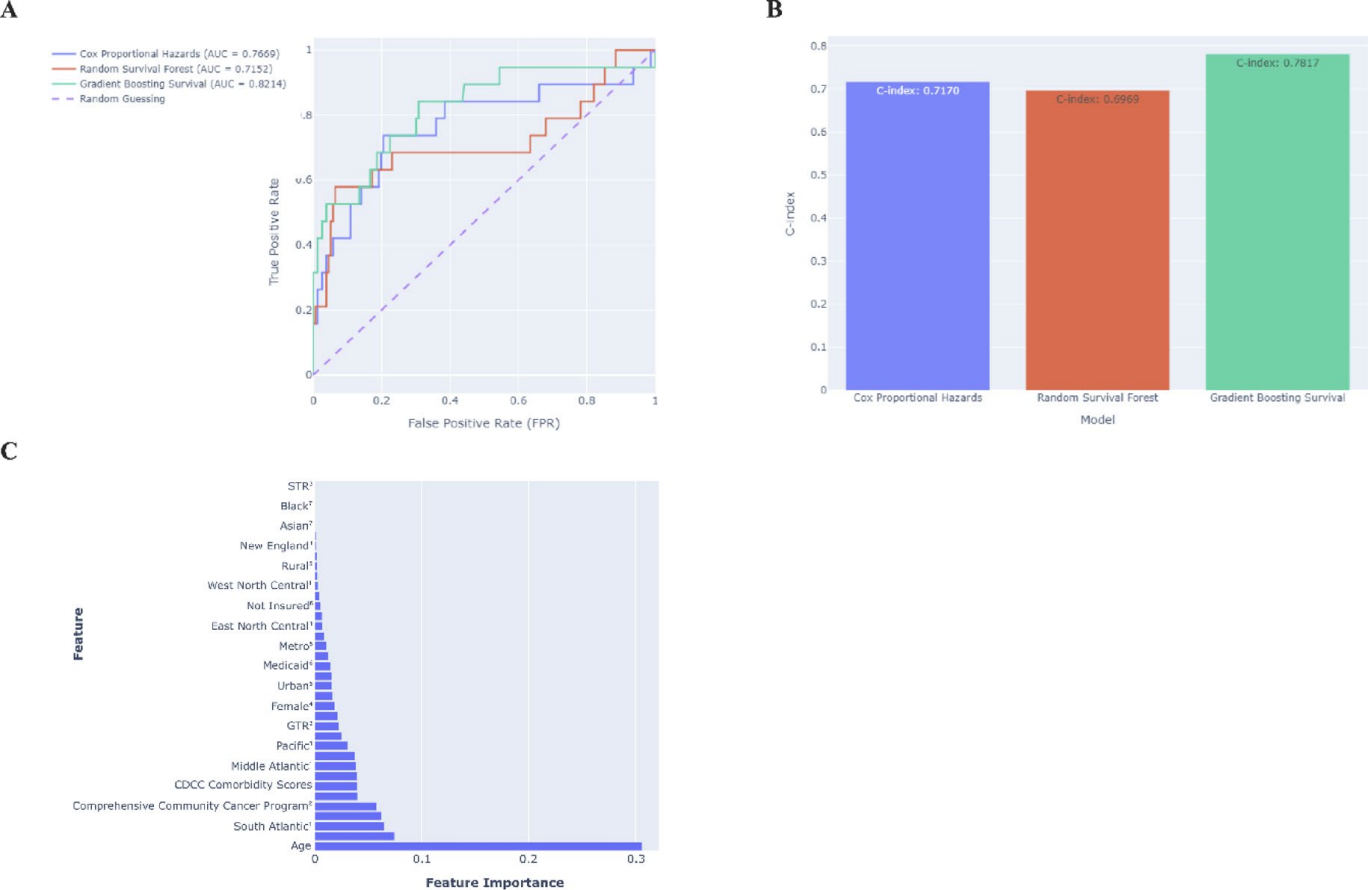
Evaluation of Survival-Based Machine Learning Models in Spinal Cord Hemangioblastoma Using (**A**) ROC Analysis, (**B**) C-Index, and (**C**) Gradient Boosting SHAP Feature Importance. *Categories*: Facility location, Facility Type, Extent of resection, Sex, Residential urbanization/rurality, Insurance Status, Race

Survival model performance was assessed using AUC from ROC curves and the C-index. The Gradient Boosting Survival model outperformed other models, achieving the highest AUC (0.8214), followed by the Cox Proportional Hazards model (AUC = 0.7669) and the Random Survival Forest model (AUC = 0.7152) (Fig. 4A). The C-index further validated these findings, with the Gradient Boosting Survival model demonstrating the highest C-index (0.7817), followed by the Cox Proportional Hazards model (0.7170) and the Random Survival Forest model (0.6969) (Fig. 4B).

SHAP analysis of the Gradient Boosting Survival model identified critical predictor of survival (Fig. 4C). Age was found to be the most influential feature. Facility type, particularly treatment at comprehensive community cancer programs, also strongly impacted survival outcomes. Additional key features included CDCC scores, facility location (e.g., South Atlantic region), and treatment modality, particularly GTR. Demographic variables such as race, sex, insurance status, and urban–rural classification had relatively lower importance.

### Gradient boosting survival calculator for spinal cord hemangioblastoma outcomes

Based on the Gradient Boosting Survival model, we developed an online calculator to predict personalized long-term mortality (over 10 years) risk among spinal cord hemangioblastoma patients. A screenshot of the calculator’s interface and a sample output are provided in Supplementary Fig. 2. This was created based on the top-performing Gradient Boosting Survival model, demonstrating the highest AUC (0.8214) and C-index (0.7817) among all evaluated models. The calculator, designed to provide personalized mortality risk estimates for patients with spinal cord hemangioblastoma based on socio-economic factors, can be accessed at the following link: https://huggingface.co/spaces/AKG47/Spine_Tumors_race

## Discussion

In this study, we used the NCDB to examine racial and socioeconomic disparities in the treatment and survival outcomes of patients with spinal cord hemangioblastoma. Our findings demonstrate how multiple factors, including race, insurance status, comorbidity burden, facility type, and treatment modality, interact to influence long-term mortality.

Among racial groups, Asian patients had the best long-term survival, followed by Black and then White patients. Black patients were more likely to have higher comorbidity scores and to fall within the lowest income quartile. In contrast, White patients were more frequently covered by private insurance, which was associated with improved survival. We also found that treatment at academic or research centers was protective, and that surgical treatment alone or combined with radiation resulted in better long-term survival compared to radiation alone. These results are consistent with prior literature in spinal oncology and support the use of individualized risk prediction tools that incorporate both demographic and treatment variables. However, in the adjusted multivariable analysis, neither race nor insurance status remained statistically significant predictors of survival. We have taken care not to overstate their influence based solely on unadjusted analyses. Rather, this suggests that their effects may be mediated through intermediary factors such as access to specialized care or treatment modality. Additionally, it underscores the importance of interpreting racial disparities cautiously and in the broader context of structural, socioeconomic, and clinical factors that collectively shape survival outcomes. For instance, the significantly higher unadjusted mortality observed among White patients, which was not sustained in the adjusted multivariable analysis, likely reflects confounding factors such as age distribution, treatment facility type, and insurance coverage patterns rather than intrinsic effects of race.

Several prior studies have reported associations between race and survival in spinal cord tumors. For example, Akinduro et al.^13^ found that Black patients had increased odds of 5-year mortality after resection of intramedullary tumors.^13^ Similarly, Elsamadicy et al.^14^ reported that Black patients in the SEER database had a higher overall mortality risk compared to White patients.^14^ Our findings align with these studies by showing significant differences in long-term survival by race. However, we observed low 30- and 90-day mortality rates across all racial groups, suggesting that disparities may be more related to long-term care access and follow-up rather than perioperative factors. Furthermore, the absence of a significant association between race and mortality in multivariable models demonstrates that race may operate through interconnected clinical variables. These results underscore the need for further research to clarify how sociodemographic factors influence outcomes and to inform strategies that promote equitable access to high-quality, longitudinal care.

The impact of race on survival may also differ depending on tumor histology. For instance, Khalid et al. found no significant racial differences in survival among patients with intramedullary astrocytomas.^15^ This highlights the possibility that racial disparities may vary between spinal tumor subtypes. Nonetheless, disparities have been documented in other outcomes such as unplanned reoperation rates and hospital costs, reinforcing the broader influence of race and socioeconomic status in spinal tumor care.^16,17^ In our study, differences in comorbidity burden and income levels may partially explain the variation in long-term survival.

Insurance coverage also played a key role in survival outcomes. Patients with private insurance had better long-term survival, while those who were uninsured or covered by Medicare had worse outcomes. These trends were reflected across racial groups. Black and Asian patients in our cohort were more likely to rely on Medicaid or be uninsured, which is consistent with previous studies. Akinduro et al. also reported lower mortality among privately insured patients with intramedullary tumors.^13^ Dasenbrock et al.^18^ similarly observed higher in-hospital mortality among Medicaid and uninsured patients undergoing surgery for spinal metastases.^18^ Additionally, Chiu et al.^7^ showed that Black and Hispanic patients were disproportionately covered by Medicaid, further reinforcing the connection between race, insurance status, and outcomes.^7^

Our findings also emphasize the importance of facility type. Patients treated at academic or high-volume centers had better survival, regardless of race. This supports the conclusions of Akinduro et al., who found improved 5-year survival for patients treated at academic centers.^13^ Similar results have been reported for other central nervous system tumors. For example, Mahato et al.^19^ found better outcomes in patients with spinal chordomas treated at academic institutions, and Battistin et al.^20^ reported similar findings in grade II gliomas.^19,20^ Although we did not observe racial differences in facility utilization in our cohort, expanding access to academic centers may help reduce disparities in outcomes.

Finally, treatment modality was a major predictor of survival. Surgery, whether alone or in combination with radiation, was associated with the best outcomes across all racial groups. In contrast, radiation alone was less effective. This aligns with prior findings by Huang et al., ^21^ who showed that both GTR and STR resulted in better survival than radiation alone in patients with intracranial and spinal cord hemangioblastomas.^21^ The lack of benefit from surgery-plus-radiation in their study may have been due to limited sample size. Our data support the conclusion that access to timely, surgical care at specialized centers is essential to optimize long-term outcomes for all patients, regardless of race or socioeconomic status.

### Limitations

This study has several limitations, primarily stemming from the constraints of the NCDB. First, the NCDB lacks genetic and molecular data, most notably the absence of VHL mutation status, which limits differentiation between sporadic and VHL-associated hemangioblastomas. This is a significant limitation, as VHL-associated cases often present earlier in life, exhibit multifocality, and carry higher recurrence risks, potentially influencing survival trajectories and treatment planning. Second, tumor level within the spinal cord (e.g., cervical, thoracic, lumbar) could not be determined due to the absence of segmental anatomical coding. This restricts our ability to evaluate how tumor location influences neurological function or prognosis. To approximate the anatomical site, we performed a subanalysis distinguishing spinal cord (C72.0) from cauda equina (C72.1) tumors; however, this remains a surrogate measure and lacks the precision needed to assess level-specific outcomes. Third, the reliability of Kaplan–Meier survival estimates beyond 10 years diminishes due to decreasing patient numbers at risk, which introduces statistical uncertainty and reduces the interpretability of long-term survival probabilities. Additionally, key clinical variables such as recurrence, progression-free survival, and neurological outcomes were not captured, limiting the ability to assess treatment effectiveness beyond OS. Fifth, the NCDB does not provide detailed zip code-level geographic data, restricting analysis of social determinants of health such as housing quality or transportation access using standardized indices (e.g., the Area Deprivation Index).^22^ Also, the small sample size of Black and Asian patients limits statistical power for subgroup comparisons. Also, median income and residential location are approximated at the ZIP code level and therefore serve only as indirect proxies of individual socioeconomic status. These measures may not capture individual variations in income, education, or access to resources. Future studies incorporating patient-reported data or geocoded composite indices may allow for more precise socioeconomic stratification. Although extent of resection (GTR vs. STR) was captured using standardized NCDB margin codes, the database does not specify whether margin status was determined intraoperatively by the surgeon or through postoperative imaging. Additionally, nuances related to surgical technique, imaging confirmation, or re-exploration procedures were not captured. As such, classification of resection extent may be subject to institutional variability in reporting practices. Another important limitation is the absence of neurological status data in the NCDB. As a result, we were unable to assess baseline or postoperative neurological function, which is a critical determinant of quality of life and treatment efficacy in spinal cord tumor patients. Additionally, the absence of actual medical expenses or cost data prevents detailed assessment of financial burdens and socioeconomic disparities, limiting our ability to evaluate how treatment affordability and out-of-pocket expenses influence patient outcomes. Lastly, as a retrospective cohort study, the analysis is inherently susceptible to selection bias, unmeasured confounding, and missing data, limiting causal inference despite multivariable adjustments. Future prospective studies incorporating genetic testing, comprehensive clinical endpoints, and more robust socioeconomic data are essential to better stratify risk and address disparities in spinal cord hemangioblastoma outcomes.

## Conclusion

Our study illustrates the importance of socio-economic and demographic factors for predicting longitudinal outcomes in spinal cord hemangioblastoma patients. Factors including Black race, uninsured status, treatment at community cancer centers, and treatment with radiation alone were found to be associated with lower long-term survival. However, these factors should be interpreted with caution, as they were not independently predictive of survival in the adjusted multivariable model. This reflects the complex interaction between sociodemographic and clinical variables as well as other confounding influences. In contrast, clinical variables including age, comorbidity burden, tumor size, and treatment characteristics emerged as stronger determinants of long-term outcomes as demonstrated in the multivariable analysis. Additionally, the specific findings relating to structural inequities bear important policy and clinical implications. Our calculator provides a practical tool for predicting individual long-term mortality for spinal cord hemangioblastoma patients considering these factors. Further investigation would also be helpful in establishing how such inequities influence other clinical outcomes, such as progression-free survival, for spinal cord hemangioblastoma patients.

## Supplementary Information

Below is the link to the electronic supplementary material.

Supplementary Material 1

## Data Availability

The data used in this study are available from the National Cancer Database (https://www.facs.org/quality-programs/cancer/ncdb ).
