# Supplementary material for: Racial and socioeconomic disparities in long term survival after surgery and radiation for spinal cord hemangioblastoma
Source: Sci Rep. 2025 Aug 21;15:30704. doi: 10.1038/s41598-025-13330-7 (PMC12371076; doi:10.1038/s41598-025-13330-7)

**Supplementary Table 1**. Multivariable Cox Proportional Hazards Model for Predictors of Overall Mortality.

| **Variable** | **HR** | **95% CIs** | ***P value*** |
| --- | --- | --- | --- |
| **Age** | 1.06 | 1.03 - 1.09 | **< 0.001** |
| **Sex** |  |  |  |
| Female | (Reference) | | |
| Male | 0.92 | 0.60 - 1.42 | 0.717 |
| **Race** |  |  |  |
| Asian | (Reference) | | |
| Black | 0.78 | 0.10 - 6.14 | 0.812 |
| White | 1.86 | 0.30 - 11.42 | 0.503 |
| **CDCC Comorbidity scores** |  |  |  |
| 0 | (Reference) | | |
| 1 | 1.77 | 0.98 - 3.18 | 0.057 |
| 2+ | 2.05 | 1.05 - 3.97 | **0.034** |
| **Primary Tumor Site** |  |  |  |
| Spinal cord | (Reference) | | |
| Cauda equina | 0.35 | 0.10 – 1.24 | 0.105 |
| **Tumor size** |  |  |  |
| ≤ 62.2mm | (Reference) | | |
| > 62.2mm | 1.52 | 0.96 – 2.42 | 0.075 |
| **Facility Type** |  |  |  |
| Academic/Research Program | (Reference) | | |
| Community Cancer Program | 2.61 | 0.61 - 11.28 | 0.198 |
| Comprehensive Community Cancer Program | 1.89 | 1.13 - 3.17 | **0.015** |
| Integrated Network Cancer Program | 0.77 | 0.37 - 1.61 | 0.486 |
| **Insurance Status** |  |  |  |
| Not issured | (Reference) | | |
| Medicaid | 0.82 | 0.24 - 2.89 | 0.763 |
| Medicare | 0.66 | 0.26 - 1.70 | 0.389 |
| Other Government | 0.65 | 0.15 - 2.84 | 0.569 |
| Private Insurance / Managed Care | 0.48 | 0.20 - 1.17 | 0.106 |
| **CROWFLY (miles)** | 1.00 | 1.00 - 1.00 | 0.567 |
| **Median Income Quartiles** |  |  |  |
| Highest Income Quartile | (Reference) | | |
| High-Middle Income Quartile | 0.99 | 0.57 - 1.71 | 0.960 |
| Middle-Low Income Quartile | 0.66 | 0.35 - 1.22 | 0.181 |
| Lowest Income Quartile | 1.62 | 0.81 - 3.23 | 0.175 |
| **Residential Location** |  |  |  |
| Metro | (Reference) | | |
| Rural | 2.64 | 0.52 - 13.31 | 0.239 |
| Urban | 2.49 | 1.26 - 4.94 | **0.009** |
| **Intervention** |  |  |  |
| Radiation alone | (Reference) | | |
| Surgery alone | 0.21 | 0.09 - 0.52 | **0.001** |
| Surgery with Radiation | 0.24 | 0.07 - 0.75 | **0.014** |
| **EOR** |  |  |  |
| GTR | (Reference) | | |
| STR | 1.64 | 0.22 - 12.04 | 0.628 |

**Supplementary Figure 1:** Temporal trends in racial distribution and surgical approach for spinal cord hemangioblastoma between 2004-2017: (A) Annual racial composition and total case volume; (B) Annual utilization of open versus MIS. A Mann-Kendall test was used to assess temporal trends.


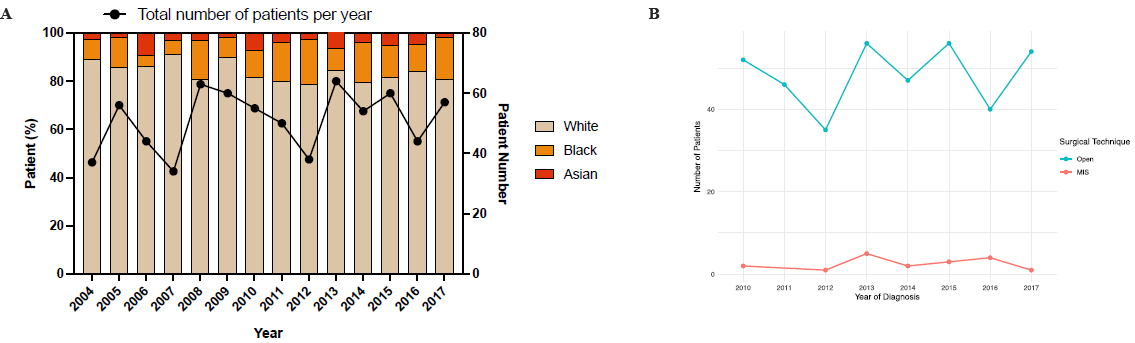


**Supplementary Figure 2:** Interface of the Gradient Boosting Long-Term Mortality Risk Predictor displaying input fields for clinical and socioeconomic variables and the resulting predicted probability of death in Patients Treated for Spinal Cord Hemangioblastomas.


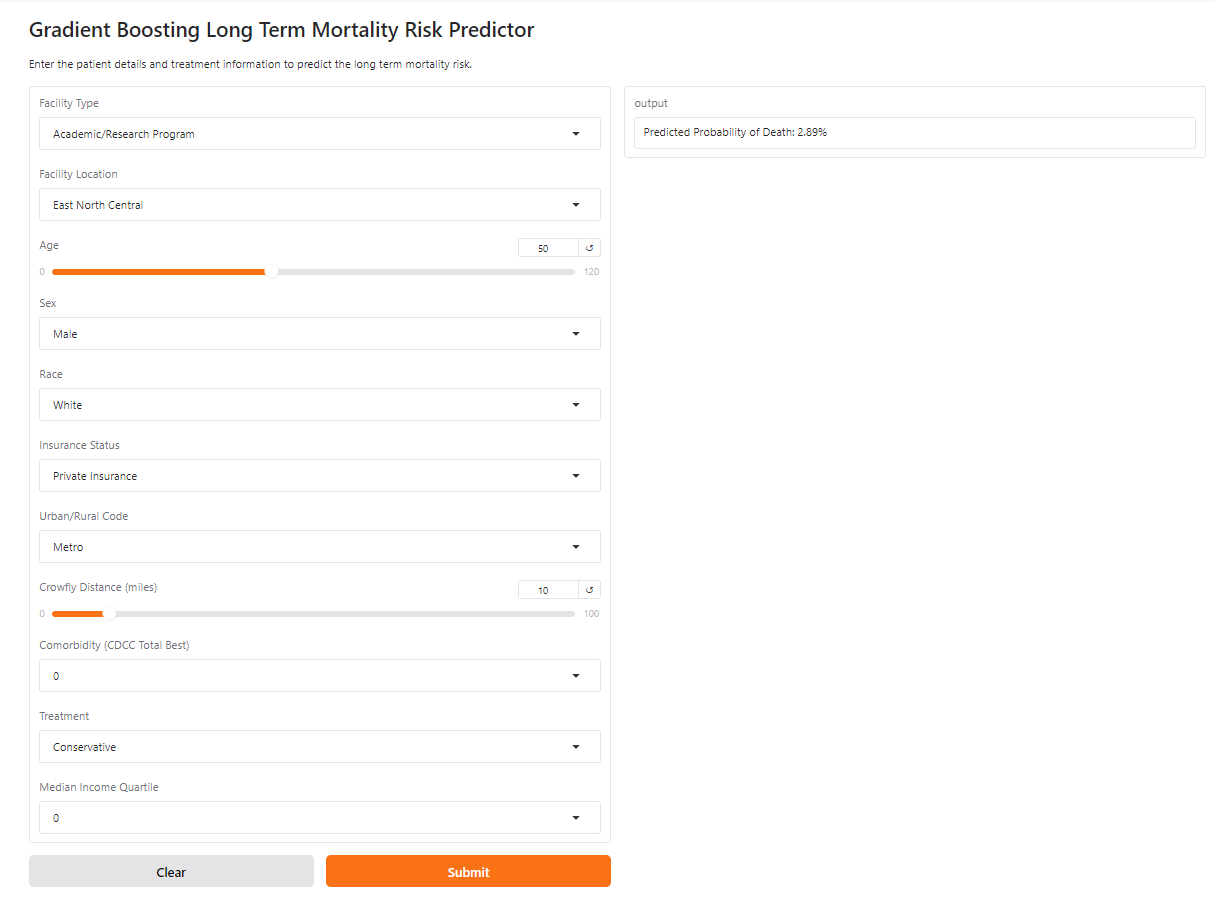

Supplement: Supplementary file 1 — Supplementary Material 1 [file 41598_2025_13330_MOESM1_ESM.docx]
